# Respiratory muscle training reduces painful swallowing and opioid use during radiation therapy for head and neck cancer: a matched pair analysis

**DOI:** 10.1186/s12885-025-13756-2

**Published:** 2025-03-12

**Authors:** Andrew D. Ray, Chi-Chen Hong, Nicolas F. Schlecht, Han Yu, Kristopher Attwood, Kent L. Nastiuk, Bryan Spinelli, Ann Marie Flores, Hillary Jacobson, Julia Fulfaro, M. Jeffrey Mador, Austin J. Iovoli, Mark K. Farrugia, Anurag K. Singh

**Affiliations:** 1 Dept. Cancer Prevention and Control, Roswell Park Comprehensive Cancer Center, Buffalo, USA; 2 Dept. Rehabilitation, Roswell Park Comprehensive Cancer Center, Buffalo, USA; 3 Dept. Biostatistics and Bioinformatics, Roswell Park Comprehensive Cancer Center, Buffalo, USA; 4 Dept. Radiation Medicine, Roswell Park Comprehensive Cancer Center, Buffalo, USA; 5 Dept. Cancer Genetics and Genomics, Roswell Park Comprehensive Cancer Center, Buffalo, USA; 6 Dept. Physical Therapy, Thomas Jefferson University, Philadelphia, USA; 7 Depts. Physical Therapy and Human Movement Sciences and Medical Social Sciences, Northwestern University, Evanston, USA; 8 Dept. Biology, Boston University, Boston, USA; 9 Dept. Medicine, Jacobs School of Medicine and Biomedical Sciences, University, Buffalo, USA; 10 Dept. Pulmonary Critical Care and Sleep, Western New York VA Medical Center, Buffalo, NY USA

**Keywords:** Oral, Performance, Respiratory muscle, Diaphragm, Pain, Quality of life, Exercise

## Abstract

**Background:**

Patients with head and neck cancer (HNC) receiving radiation therapy (RT) are at increased risk for symptoms of oral mucositis (OM), opioid use, and declines in physical function, outcomes that contribute to increased morbidity and mortality. The study objective was to determine the effects of respiratory muscle training (RMT) on OM and opioid use, as well as functional performance in patients with HNC receiving RT with or without concurrent chemotherapy (CCRT).

**Methods:**

Patients aged ≥ 18 years of age with stage I to IV HNC being treated with RT or CCRT receiving a home-based respiratory muscle training (RMT) (*n* = 20) were compared to a 5:1 matched historical group (*n* = 100) who did not receive RMT. RMT was delivered using the commercially available Power Lung AireStream device (Houston, TX) via a standardized home-based inspiratory and expiratory muscle-training program requiring ~ 20–30 min/day, five days per week, with a progressively increasing workload. Primary endpoints collected from all patients included changes in OM symptoms and use of opioids for pain control following start of RT. Secondary outcomes collected on RMT patients included respiratory muscle strength and functional performance (Six-Minute Walk Test, 6MWT; Short Physical Performance Battery, SPPB). All measures were assessed before and within 1–2 weeks following a standard 7-week RT regimen.

**Results:**

RMT reduced the impact of self-reported swallowing soreness (*p* = 0.032), eating soreness (*p* = 0.036), and opioid use (*p* = 0.015). RMT maintained inspiratory muscle strength (+ 0.6 ± 18 cmH2O, *p* = 0.87), expiratory muscle strength (+ 0.7 ± 12.7 cmH2O, *p* = 0.197), and improved the 6MWT (+ 20 ± 39.9 m, *p* = 0.025), with no change in the SPPB total score (*p* = 0.262).

**Conclusions:**

RMT is a low-cost intervention that is easy to perform among patients undergoing RT/RTCC for HNC and is likely to reduce OM pain/symptoms and opioid, as well as to preserve respiratory muscle strength and physical function during cancer treatment.

**Trial registration:**

Not applicable. This was a matched retrospective cohort study not registered as it was a nonrandomized trial with a historical control group.

## Introduction

The United States is estimated to have 58,450 new cases and 12,230 new deaths from head and neck cancer (HNC) in 2024^1^. At least 60% of these patients present with locally advanced, non-metastatic disease that is commonly treated with radiation therapy (RT) or concurrent chemoradiation therapy (CCRT) [2]. Acute and chronic pain during and following RT/CCRT for HNC is prevalent with 98% of patients developing oral mucositis (OM) [3–5], and more than 60% of these patients reporting severe OM pain during treatment [3–5]. Importantly, OM is associated with hospitalization, feeding tube placement, weight loss, and reduced functional status [5, 6]. It is also estimated approximately half of patients with HNC being treated with RT/CCRT require opioids for pain control during treatment [7]. Opioid use requires treating physicians to balance the goal of optimal pain control with the risk of addiction and abuse. Non-pharmacological interventions that can potentially improve pain management and reduce opioid use, such as exercise, may be beneficial in these patients [8], although whole-body exercise programs during cancer treatment may not be possible for all patients.

Patients with HNC are also at risk of losing lean mass [9], changes that influence functional performance, physical activity, swallowing, and pain [9–12]. Recently, treatment for HNC has been shown to influence inspiratory and expiratory muscle performance as diaphragm strength, mobility, and thickness all decline with treatment [13, 14], which can influence symptoms (i.e., dyspnea and fatigue), cardiorespiratory fitness, and morbidity and mortality [15]. Expiratory muscle weakness also influences swallowing performance, aspiration, and pneumonia risk [13, 14]. Respiratory muscle training (RMT) programs improve respiratory muscle strength, cardiorespiratory fitness, as well as dysphagia, and swallowing safety in other patient populations [16–20]. Despite the benefits of RMT, few studies have been performed in patients with HNC during treatment. One small study suggests that inspiratory muscle training at a lower training resistance is feasible during CCRT, however, it could not prevent the decline in inspiratory muscle strength and the six-minute walk test (6MWT) [21]. To our knowledge, no studies in patients with HNC have reported on OM or opioid use as well as the effects of a progressively increasing inspiratory and expiratory muscle training program during RT or CCRT.

The primary objective of this study was to examine the benefits of a home-based inspiratory and expiratory muscle training program for patients receiving RT or CCRT for HNC. Because maintaining muscle mass during cancer treatment is associated with less OM pain in HNC [22], we further hypothesize maintaining activity of the swallowing musculature, via RMT, will help to reduce painful swallowing and translate into less need for pain control with opioids. Secondarily, RMT during RT/CCRT was postulated to improve functional performance outcomes.

## Methods

This study was performed under a protocol approved by the Roswell Park Comprehensive Cancer Center Institutional Review Board (IRB; study number: EDR-103707) and conducted in accordance with the Transparent Reporting of Evaluations with Nonrandomized Designs (TREND) reporting guidelines [23] and the current Declaration of Helsinki [24]. A waiver of consent was obtained from the IRB due to the retrospective nature of this study.

### Study Population

Patients aged ≥ 18 years with a diagnosis of stage I-IV HNC scheduled to receive RT alone or CCRT between May 2022 and May 2023 were eligible for inclusion. After May 2022, all HNC patients were offered the optional home-based RMT as part of standard of care. During this one-year period, a total 50 HNC patients were scheduled for RT or CCRT at Roswell Park and offered the option to receive home-based RMT by clinical staff.

### Study design

This was a matched retrospective cohort study evaluating the effects of home-based RMT on OM outcomes, opioid use, and physical function in patients with HNC. HNC patients who received home-based RMT as part of standard clinical care at our institution after May 2022 were compared to similar HNC patients who did not receive home-based RMT. In the current analysis, patients who received home-based RMT between May 2022 to May 2023 were matched to historical patients treated at the same Radiation Medicine Clinic between May 2018 and May 2022 using 5:1 matching based on age, sex, race, clinical stage, and body mass index. Exact matching was used for categorical variables and nearest neighbor matching was used for the continuous variables. Matching was implemented using the MatchIt package in R [25]. The statistician performing the matching was blinded to OM, opioid use, and functional outcomes. A within group pre/post-design was used to examine the effects of RMT on functional outcomes.

### Clinical and demographic characteristics

As part of routine care, all patients completed a staging workup with computed tomography (CT) of the head and neck with contrast and/or positron emission tomography-computed tomography (PET/CT). Patients were treated with intensity-modulated radiation therapy (IMRT; 70 Gy/35 fractions to the primary tumor, 56 Gy/35 fractions to elective lymph nodes) with or without CCRT, as previously described [26]. The most prescribed regimen was cisplatin 100 mg/m^2^ on days 1, 22, and 43 of radiotherapy or cisplatin 30 to 40 mg/m^2^ weekly.

Prior to and during treatment, all patients received educational materials regarding oral hygiene, and hydration. Patients were encouraged to gargle with a saline/baking soda mouthwash rinse as many times as possible per day (e.g., 20 times) and use a compounded elixir of diphenhydramine, xylocaine, and antacid in a 1:1:1 ratio 4 times per day for pain. To minimize OM, home humidification and oral rinses were recommended, and gabapentin was prescribed at the beginning of treatment [27]. Upon development of symptomatic OM, either doxepin or diphenhydramine-lidocaine-antacid (DLA) mouthwash was initiated. When pain was no longer adequately controlled via this regimen, alternating doses of ibuprofen and acetaminophen were recommended. Patients were instructed to take 400 mg of ibuprofen and 4 h later, take 1,000 mg of acetaminophen until the maximum recommended daily dose of acetaminophen (3,000 mg/day) was reached. In the last weeks of treatment, when adequate pain relief was difficult, methadone (2 mg three times a day) was used to supplement the above regimen.

A medical chart review was performed on all patients referred for RMT and matched non-RMT patients to collect information baseline and post-treatment information on demographics, opioid prescriptions, treatment type, dose, and duration, as well as body weight, height, and body mass index (BMI m/kg^2^).

### Hone-based RMT

Interested patients were referred to the physical therapy clinic for implementation prior to start of RT and again with 1–2 weeks upon completion of RT. Patients were given a home-based RMT program to be performed concurrently with their 7-week RT regimen. The home-based RMT intervention was delivered via a standardized inspiratory and expiratory muscle-training program developed in our previous work [28]. Patients were instructed to perform three sets of 15 inspiratory and expiratory breaths per day, five days per week, beginning at a moderate resistance level (5/10) based on the Modified Borg Scale (0 to 10). Resistance was progressively increased each week to maintain a perceived exertion level of 5–7/10 on the scale, ensuring a gradual progression in training intensity. Training was performed with the commercially available Power Lung AireStream device (Houston, TX). The RMT program was administered by a licensed physical therapist.

### Primary outcomes

#### Self-reported oral Mucositis and Opioid Use Data from Medical records

Patients were evaluated weekly by the Radiation Medicine clinic team through physical examination and patient-reported responses to a modified OMWQ survey [29]. The OMWQ is a valid and reliable survey assessing patient well-being and function and includes questions assessing patients’ mouth and throat soreness and its impact on daily functioning [29]. All questions used a Likert-type response format. Questions assessed overall health and quality of life in past week (scored on a 7-point scale from Very poor to Excellent); amount of mouth and throat soreness (2 questions, one scored on a scale from 0 to 10 and another using a Likert scale: none, a little, moderate, quite a lot and extreme soreness); and how much the soreness limited activities (scored on a 5-point scale from “not limited” to “unable to do”) including, (1) sleeping, (2) swallowing, (3) drinking, (4) eating, and (5) talking.

Pain medications, dosages and OMWQ scores were recorded weekly and contemporaneously in both the electronic medical records and a departmental clinical database.

### Secondary outcomes

Functional outcomes were only measured in the RMT group (*n* = 24) and included the following:

#### Respiratory muscle strength

Maximal inspiratory muscle pressure (MIP) and maximal expiratory muscle pressure (MEP) were measured with a handheld digital manometer (Micro Respiratory Pressure Meter, CareFusion, Yorba Linda California) according to American Thoracic Society (ATS) guidelines [30].

#### Aerobic capacity

The six-minute walk test (6MWT) was used to estimate cardiopulmonary endurance [31] and was performed according to ATS guidelines [32].

#### Lower extremity strength

The 30-second sit-to-stand test (30STS) is a validated measure of lower body strength [33] and physical functioning in patients with cancer [34, 35]. Patient were instructed to complete as many full sit-to-stands as possible within 30 s.

#### The short physical performance battery (SPPB)

The SPPB is a short battery of performance tests of lower extremity functioning (balance, gait speed, and strength) [36] and is associated with muscle mass, risk of falls, and mortality [37]. Briefly, gait speed required patients to walk 4 m at their usual pace. Standing balance required patients to maintain their feet together, semi-tandem, and tandem for 10 s each. The 5 sit-to-stand (STS) tests required patients to rise from a standard height chair five times as quickly as possible with their arms across their chest. Scores range from zero (worst) to 4 (best) for each test [36]. The sum of the three SPPB components comprised the final SPPB score (range 0 to 12). A score of 12 indicates the highest degree of lower extremity functioning [36].

### Statistical analysis

Data distributions for continuous variables were summarized as medians and inter-quartile ranges (IQR). Differences between RMT and matched non-RMT patients were examined using Wilcoxon rank-sum tests for continuous variables and Fisher’s exact tests for categorical variables. Change in OM pain scales were calculated by considering measurements in week 1 of RT as the baseline. The number of usages of opioid containing medications (i.e., Lortab, Fentanyl, Methadone, Oxycodone/OxyContin, Venlafaxine) were converted to morphine milligram equivalents for each patient at each week. The time-averaged difference between the two groups in their change from baseline in week 2 to 7 was examined using linear mixed model with RMT as the fixed effect and time since start of RT as the random effect. The model fit was assessed using the restricted maximum likelihood (REML) method as implemented in R lme4 package. The average trajectories in the two groups were visualized using locally estimated scatterplot smoothing (LOESS). All tests were two sided and *p* < 0.05 was considered statistically significant.

## Results

**Fig. 1:**
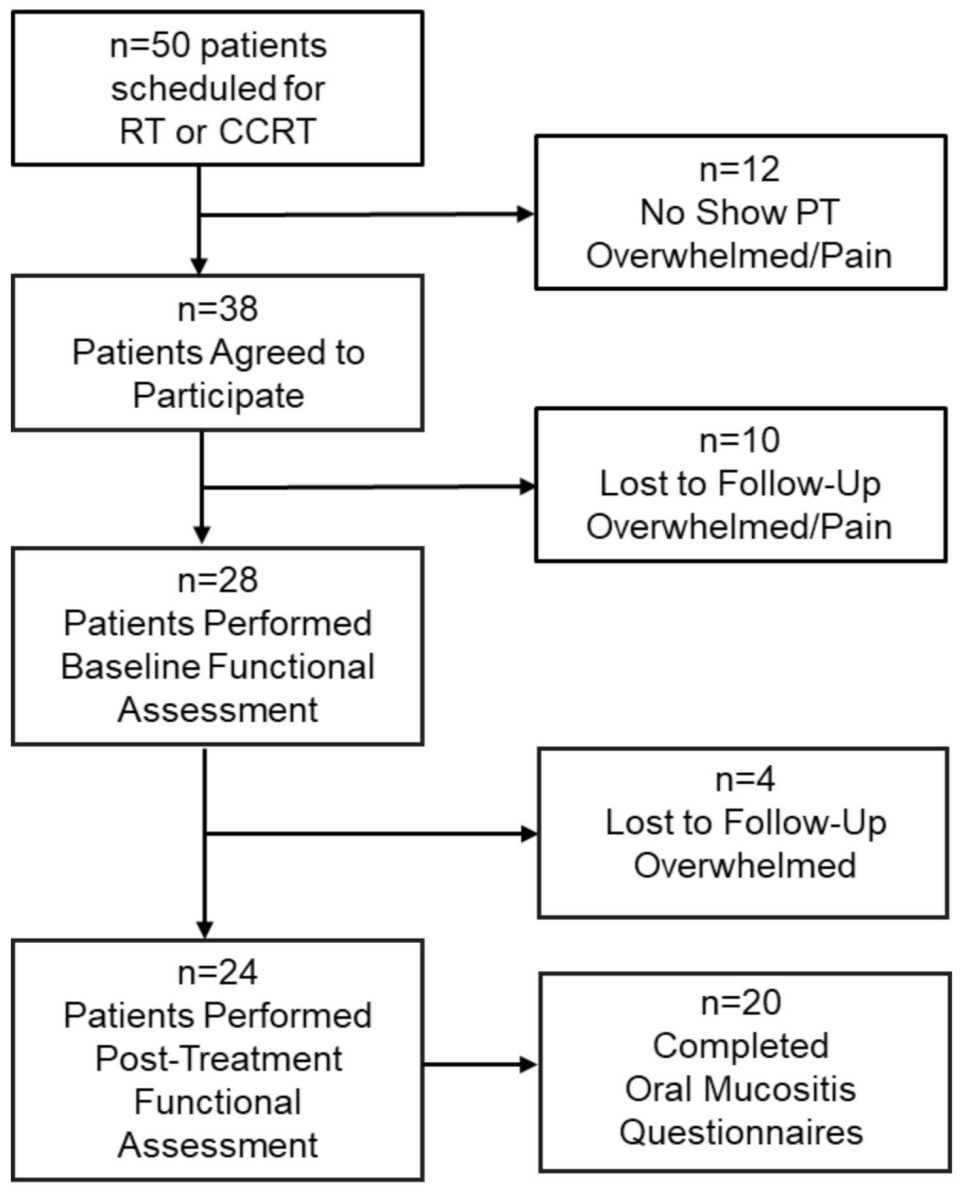
Patient flow diagram. A total of 50 HNC patients were treated with RT or CCRT in the Radiation Medicine between May 2022 and May 2023, of which 38 agreed to receive optional home-based RMT as part standard of care. Four patients discontinued RMT following the baseline evaluation without providing a reason, resulting in 24 patients with both pre- and post-RMT assessments. Of these, 20 patients completed the OM symptoms scales and were matched 5:1 to a historical non-RMT group for analysis of primary endpoints (OM symptoms and use of opioids for pain control)

Of the 50 HNC patients in the Radiation Medicine clinic between May 2022 and May 2023, during the study period, more than half (*n* = 38) agreed to receive home-based RMT; but only 28 attended their physical therapy initial evaluation (Fig. 1). The primary reason for declining RMT was feeling overwhelmed by existing medical appointments. Among the 28 who initiated RMT, four withdrew after their baseline assessment, citing similar concerns. Ultimately, 24 patients completed the home-based RMT intervention. For the oral mucositis (OM) analysis, an additional four patients were excluded due to missing OM data. At our facility, the OMWQ is routinely administered during treatment, but it is unclear why these patients did not provide responses. The 20 patients with OM data were then matched 5:1 to a retrospective cohort of 100 HNC patients who were also treated in the Radiation Medicine clinic and provided responses OM analysis. There were no differences in baseline demographics between the RMT (*n* = 20) and the matched non-RMT (*n* = 100) groups (Table 1). At the completion of treatment, there were no differences (*p* = 0.24) in the median (IQR) loss of body weight in the RMT [-4.9 kg (1.7, 8.8)] vs. non-RMT [-6.7 kg (3.1, 11.6)] groups.

**Table 1:** Patient demographics

Variable	Home-Based RMT HNC Patients	Non-RMT HNC Patients	*p*-value^1^
**#**	20	100	
**Age**,** years**,** Median (IQR)**	60.6 (52.3, 68.8)	58.7 (54.9, 66.9)	0.92
**Weight**,** kg**,** Median (IQR)**	95.5 (80.4, 104.5)	87.6 (72.7, 100.6)	0.50
**BMI**,** Kg/m**^**2**^, **Median (IQR)**	30.5 (26.1, 32.1)	28.7 (24.5, 32.1)	0.50
**Sex**,** N (%)**			
Male	17 (85)	85 (85)	1.0
Female	3 (15)	15 (15)	
**Race**,** N (%)**			
Black	1 (5)	5 (5)	1.0
White	19 (95)	95 (95)	
**Stage**,** N (%)**			
I	7 (35)	35 (35)	1.0
II	7 (35)	35 (35)	
III	3 (15)	15 (15)	
IV	3 915)	15 915)	
**Site**,** N (%)**			
Larynx	7 (35)	22 (22)	0.86
Lateral Neck	2 (10)	12 (12)	
Lip/Oral Cavity	2 (10)	12 (12)	
Nasal Cavity/Sinus	0 (0)	1 (1)	
Pharynx	9 (45)	51 (51)	
Salivary Gland	0 (0)	1 (1)	
Other	0 (0)	1 (1)	
**Treatment**,** N (%)**			
CCRT	11 (55)	60 (60)	0.61
ICT + CCRT	1 (5)	2 92)	
RT	5 (25)	17 (17)	
Surgery CCRT	2 (10)	9 (9)	
Surgery RT	1 (5)	12 (12)	
**OMWQ**			
Overall Health	82.1 (2.9)	81.2 (15.9)	0.85
MTS	0.35 (0.7)	0.48 (0.7)	0.34
Swallowing	0.20 (0.6)	0.22 (0.6)	0.63
Drinking	0.15 (0.5)	0.18 (0.6)	0.92
Eating	0.20 (0.6)	0.24 (0.7)	0.82
Talking	0.20 (0.5)	0.35 (0.9)	0.62
Sleeping	0 (0)	0.23 (0.6)	0.08
Overall MTS Soreness Rating	10.4 (18.3)	14.7 (22.4)	0.93
**Opioid Use**			
Opioid Medications, MME	0.65 (0.5)	0.65 (0.5)	0.51

^1^Differences between home-based RMT and matched non-RMT patients were examined using Wilcoxon rank-sum tests for continuous variables and Fisher’s exact tests for categorical variables. Abbreviations: OM, oral mucositis; BMI, Body mass index; CCRT, concurrent chemotherapy with radiation therapy; ICT, induction chemotherapy; RT, Radiation therapy; OMWQ-HN, the Oral Mucositis Weekly Questionnaire–Head and Neck Cancer; MTS, Mouth and throat soreness; MME, morphine equivalents

### Effect of home-based RMT on oral mucositis

**Fig. 2:**
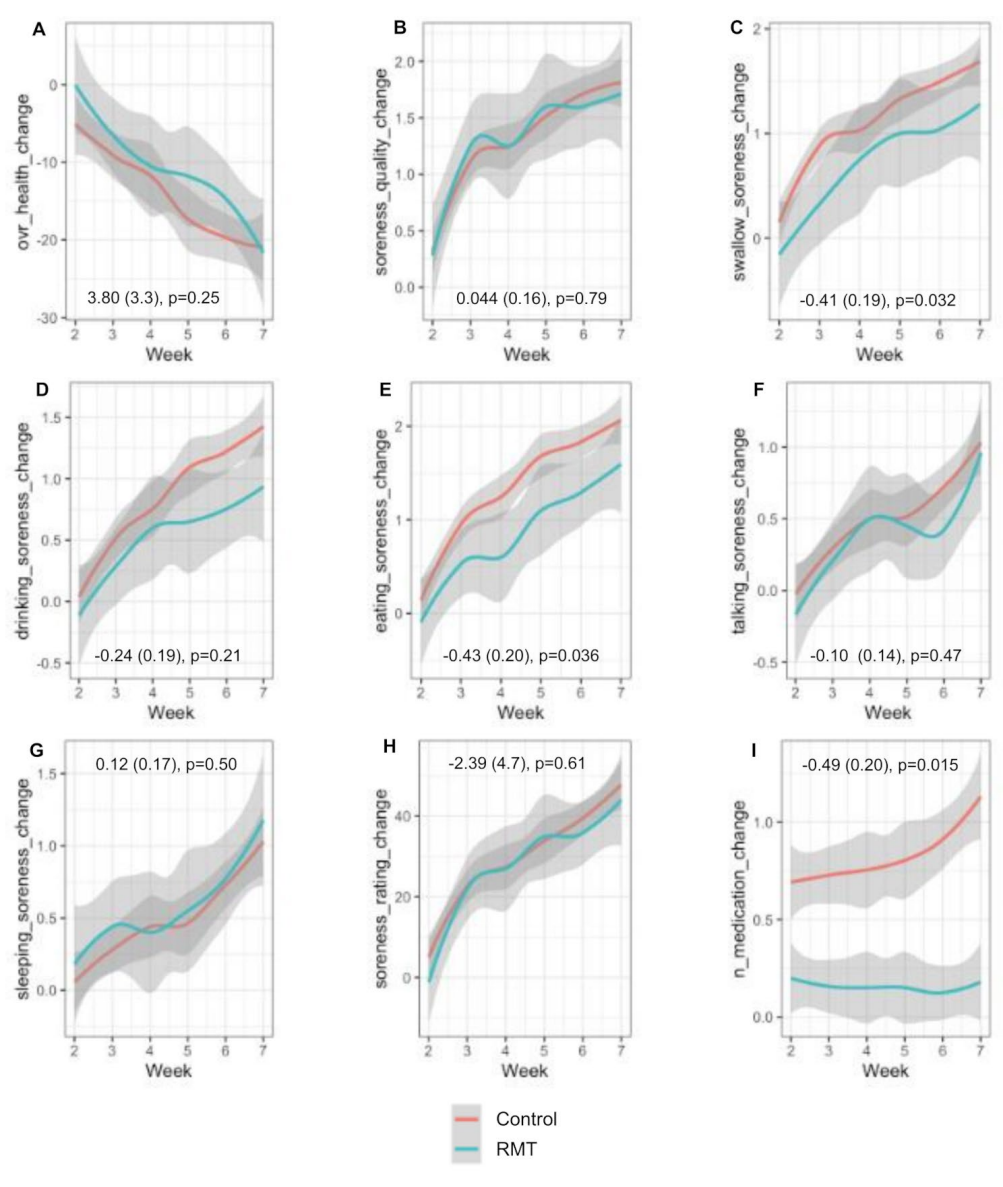
Oral mucositis and opioid use. The panels in Fig. 2 display overall health **(A)**, and mouth and throat soreness **(B)**, as well as how Mouth and Thorat Soreness limited swallow soreness **(C)**, drinking soreness **(D)**, eating soreness **(E)**, talking soreness **(F)**, sleeping soreness **(G)**, and overall mouth and throat soreness **(H)** over the previous 24 h, as well as the change in morphine equivalents **(I)** over the 7-week treatment. OM pain and opioid use during the first seven weeks were analyzed using linear mixed model. The Week 1 scores were considered as baseline. The trajectories estimated by Lowess estimator. Data represents the estimate (SE) and p-values

There were no differences between the RMT (*n* = 20) vs. matched pair non-RMT (*n* = 100) groups in OMWQ at baseline (Table 1). Figure 2 shows there was a consistent worsening across all OM soreness scales from the OMWQ for both groups. However, patients receiving RMT had a significantly smaller increase in measures of impact of OM soreness on swallowing (-0.41 ± 0.19, *p* = 0.03) and eating (-0.43 ± 0.2, *p* = 0.04) compared to the matched pair non-RMT patients.

### Effect of home-based RMT on opioid drug use

There were no between RMT (*n* = 20) vs. Matched Pair (*n* = 100) group differences in the use of opioid containing medications expressed in morphine milligram equivalents at baseline (Table 1). A significant difference was observed in the amount of morphine equivalents used during treatment as the RMT patients were less likely to receive prescribed opioid medications compared to the matched non-RMT patients (-0.48 ± 0.2, *p* = 0.01) (Fig. 2).

### Secondary outcomes in RMT patients

There were no significant differences in baseline characteristics between the (*n* = 20) RMT cohort compared to the 8 HNC patients that were referred to RMT but withdrew or were missing OM measurements. There was only a tendency for mean (SD) inspiratory muscle strength to be stronger in the (*n* = 20) RMT cohort vs. the 8 patients that withdrew or excluded (69.7 ± 22.2 cmH2O, vs. 48.2 ± 26.2, *p* = 0.060). Within group changes pre- to post-RMT are presented in Table 2.

**Table 2:** Pre- to Post-RMT changes in the home-based RMT Cohort. Means (SD)

Functional Outcomes	Pre-RMT *n* = 24	Post-RMT *n* = 24	Delta	*p*-value^1^
Weight (kg)	95.7 (37.4)	86.2 (28.7)	-6.3 (7.1)	0.0017*
BMI (kg/m2)	29.3 (9.3)	27.2 (7.8)	-1.8 (2.0)	0.0003*
MIP (cmH2O)	68.4 (21.6)	69.2 (21.1)	0.6 (17.6)	0.87
MIP (% pred)	71.3 (22.0)	71.9 (21.4)	0.6 (17.6)	0.89
MEP (cmH2O)	73.3 (21.8)	75.2 (23.0)	0.7 (12.7)	0.20
MEP (% pred)	63.4 (18.1)	65.0 (19.3)	0.6 (10.2)	0.77
5 x STS (sec)	9.2 (2.5)	8.5 (2.3)	-0.7 (1.4)	0.024*
30 s STS (reps)	15.9 (4.5)	15.7 (5.0)	-0.3 (2.5)	0.62
4 m gait time (sec)	3.74 (0.6)	3.64 (0.7)	-0.2 (0.4)	0.023*
SPPB (total points)	11.6 (0.8)	11.7 (0.8)	1.4 (3.5)	0.26
6MWT (m)	434 (84)	454 (88)	20.0 (39.9)	0.025*
6MWT (% pred)	74.6 (22.6)	78.7 (15.9)	5.1 (17.7)	0.34

Functional outcome data are presented for the *n* = 24 patients completing the home-based RMT program ^1^. Differences pre- to post-RMT/treatment were examined with a paired t-tests. Abbreviations: BMI, Body mass index; MIP, Maximal inspiratory muscle strength; MEP, maximal expiratory muscle strength; 5xSTS, 5 times sit to stand tests; 30 s STS, 30 s sit to stand test; SPPB, Short physical Performance Battery; 6MWT, Six-minute Walk test

#### Body Weight

On average, the 24 patients completing the RMT program lost body weight (-6.3 ± 7.1 kg, *p* = 0.0007) and showed a decline in BMI (-1.8 ± 2.0 kg/m2, *p* = 0.0003).

#### Respiratory muscle strength

There was no change in mean MIP (delta 0.6 ± 17.6 cmH_2_O, *p* = 0.87) or MEP (0.7 ± 12.7 cmH_2_O, *p* = 0.78) at the completion of RT.

#### Functional performance

The mean distance (SD) covered on the 6MWT (delta, 20.0 ± 39.9 m, *p* = 0.025), time to complete the 5-STS (delta, -0.7 ± 1.4 s, *p* = 0.024) and 4-meter gait times (delta, -0.2 ± 0.4 s, *p* = 0.023) improved from the beginning to the end of RT. However, total SPPB scores (1.4 ± 3.5, *p* = 0.155) and the 30STS (-0.3 ± 2.5 reps, *p* = 0.615) did not change post-RT.

## Discussion

Pain during and following treatment is common as most HNC patients develop OM [3–5] that is associated with opioid use and worse functional status [5, 6]. The current study demonstrates RMT during RT or CCRT for HNC did not influence mouth soreness ratings, however it did reduce the impact of OM soreness on self-reported swallowing soreness and eating soreness, as well as the amount of opioid use during treatment. RMT was also observed to maintain respiratory muscle strength and improve functional performance at the completion of treatment, in contrast to previous reports demonstrating a treatment-related decline in functional performance in the absence of RMT [38].

This may be the first study to show a relationship between RMT with OM pain and/or opioid use in general. The only other known rehabilitation study to show a reduction in pain during CCRT for patients with HNC included jaw mobility and stretching exercises designed to minimize trismus-induced pain [39]. The only other reports to show a relationship between breathing exercise with oral pain include a study in children (*n* = 35) using a bubble blower to reduce oral mouth pain when performed prior to dental work [40] and a case study showing virtual reality breathing exercises reduced oral pain in a 40-year-old male who underwent a mandibulectomy for left buccal mucosa carcinoma [41]. The underlying mechanisms describing how RMT or breathing exercises can reduce OM pain are not fully understood, but may include peripheral and central mechanisms [42]. For example, deep breathing exercises, similar to RMT, have been shown to release edogenous opiods, endorphins, and serotonin [42, 43]. RMT can also reduce stress and anxiety and therefore pain sensitivity [44, 45], and potentially a reduction of pain thresholds as seen with whole body exercise [42, 46].

Although our findings are preliminary, they are clinically relevant and meaningful, as others [22] have reported an inverse association between lean muscle mass with acute OM pain in patients undergoing CCRT following oral cancer resection. We hypothesize RMT may have reduced the need for opioid pain medications because it maintained activity of the swallowing musculature [47], as opposed to a decrease in swallowing muscle activity or size with treatment. This mechanism is supported by work in healthy adults showing breathing exercises including RMT, activate the submental muscles of the jaw involved in swallowing [47]. Maintaining activity of the swallowing musculature, joints, and ligaments in the mouth and throat potentially reduces local pressure pain and central sensitization [42, 48]. Importantly, improvements in swallowing and eating might be reduced even without changes in OM pain, suggesting tailored exercises that maintain swallowing muscle activity could be beneficial [8]. However, this is all speculative and testing these hypothesized theories require additional investigation.

The ability to preserve inspiratory and expiratory muscle strength throughout 7-weeks of RT/CCRT treatment contrasts with others who report a decrease in respiratory muscle strength [13, 21]. Vira et al. [21], demonstrated inspiratory muscle training during CCRT (*n* = 10) was feasible and maintained diaphragm thickness and expiratory muscle strength, however significant declines were seen in diaphragm mobility and strength. A case study by D’Souza et al. [13] showed expiratory muscle training could not prevent a decline in respiratory muscle strength in a 60 year old male receiving CCRT for a stage IV HNC. Potential explanations describing why respiratory muscle strength may not change or decline during treatment include the lower training resistances, small sample sizes, and pain [49], as post-treatment expiratory muscle training, when pain is reduced, in patients with HNC (*n* = 26) experiencing radiation associated aspiration improved expiratory muscle strength and swallowing safety [50].

Patients performing RMT significantly improved the distance covered on the 6MWT albeit the change was below the minimal clinically important difference of 26–30 m, the time to complete the 5 x sit-to-stand test, and gait speed, while observing no changes in the 30 s sit-to-stand test and total SPPB points pre- to post-treatment. The results of this study compared to those reported by Samuel et al. [51], who implemented an 11-week whole-body aerobic and resistance training program during the 7 week period, continuing for 4 additional weeks post-RT/CCRT in patients with HNC. Samuel et al., observed a decrease in the distance covered on the 6MWT immediately following treatment (∆=-5 m), which later reversed 1-month post-treatment (∆= 36 m).In contrast, the current RMT study demonstrated an improvement in the 6MWT distance (∆= 21 m) immediately post-treatment. However, these findings differ from those of Vira et al., who found inspiratory muscle training during CCRT could not prevent declines in the 6MWT [21]. It is hypothesized that RMT may alleviate treatment-related symptoms such as dyspnea and fatigue, potentially increasing daily activity levels [28, 52]. These align with previous studies suggesting that small increases in daily activity (+ 1,000 steps/day) can enhance functional capacity, and reduce frailty [53].

### Limitations

The primary limitations of this study include the small sample size of the intervention group and the retrospective study design, which lacks randomization. These factors highlight the need for confirmation of findings through a prospective, randomized clinical trial. Additionally, pain experienced by patients immediately post-treatment may have affected respiratory muscle strength testing, potentially underestimating the improvements achieved through respiratory muscle training (RMT). Future trials would also benefit from incorporating measures of daily physical activity, swallowing function, and oral mucositis biomarkers to provide a more comprehensive evaluation of the intervention’s impact.

## Conclusion

In summary, this study suggests that RMT may be a promising intervention during RT/CCRT to reduce swallowing discomfort, opioid use, and prevent a decline in function. This is a clinically relevant finding because RMT may be more implementable than whole body exercise, providing the justification to perform a more definitive prospective, randomized clinical trial to assess the efficacy of RMT in patients with HNC. Non-pharmacological approaches to managing pain during cancer treatment may reduce risks of addiction and overdose and reduce limitations in daily activities that reduce quality of life. Ultimately, our findings reinforce the need for tailored exercise programs to optimize physical functioning and clinical outcomes in head and neck cancer patients.

## Data Availability

The data underlying this article cannot be shared publicly for the privacy of individuals that participated in the study. The data are available from the corresponding author upon reasonable request.

## Abbreviations

3-STS: 30-second Sit-To-Stand
5-STS: 5 x Sit-To-Stand
6MWT: Six-Minute Walk Test
ATS: American Thoracic Society
BMI: Body Mass Index
CCRT: Concurrent Chemoradiation Therapy
CT: Computed Tomography
DLA: Diphenhydramine-Lidocaine-Antacid
Gy: Grey
HNC: Head and Neck Cancer
IMRT: Intensity-Modulated Radiation Therapy
IQR: Inter-Quartile Ranges
MEP: Maximal Expiratory Pressure
MIP: Maximal Inspiratory Pressure
OM: Oral mucositis
OMWQ: Oral Mucositis Weekly Questionnaire–Head and Neck Cancer
PET: Positron Emission Tomography
REML: Restricted Maximum Likelihood
RMT: Respiratory Muscle Training
RT: Radiation Therapy
SD: Standard Deviation
SPPB: Short Physical Performance Battery
TREND: Transparent Reporting of Evaluations with Nonrandomized Designs
